# Cryo-Electron Tomographic Structure of an Immunodeficiency Virus Envelope Complex In Situ

**DOI:** 10.1371/journal.ppat.0020083

**Published:** 2006-08-25

**Authors:** Giulia Zanetti, John A. G Briggs, Kay Grünewald, Quentin J Sattentau, Stephen D Fuller

**Affiliations:** 1 University of Oxford, Division of Structural Biology, Wellcome Trust Centre for Human Genetics, Henry Wellcome Building for Genomic Medicine, Headington, United Kingdom; 2 Department of Chemistry and Biochemistry, Ludwig-Maximilians-Universität, Munich, Germany; 3 Department of Molecular Structural Biology, Max Planck Institut für Biochemie, Martinsried, Germany; 4 The Sir William Dunn School of Pathology, Oxford University, Oxford, United Kingdom; King's College, London, United Kingdom

## Abstract

The envelope glycoprotein (Env) complexes of the human and simian immunodeficiency viruses (HIV and SIV, respectively) mediate viral entry and are a target for neutralizing antibodies. The receptor binding surfaces of Env are in large part sterically occluded or conformationally masked prior to receptor binding. Knowledge of the unliganded, trimeric Env structure is key for an understanding of viral entry and immune escape, and for the design of vaccines to elicit neutralizing antibodies. We have used cryo-electron tomography and averaging to obtain the structure of the SIV Env complex prior to fusion. Our result reveals novel details of Env organisation, including tight interaction between monomers in the gp41 trimer, associated with a three-lobed, membrane-distal gp120 trimer. A cavity exists at the gp41–gp120 trimer interface. Our model for the spike structure agrees with previously predicted interactions between gp41 monomers, and furthers our understanding of gp120 interactions within an intact spike.

## Synopsis

HIV (human immunodeficiency virus) causes AIDS (acquired immunodeficiency syndrome) that is responsible for approximately 50 million infections since its first description in 1981. Antiviral therapies have made enormous progress, but a vaccine remains essential and yet elusive. The phenotypic variability of the virus (particle size varies by 3-fold) makes a structural approach difficult. Common virus surface components must be maintained to allow attachment to and penetration of host cells for infection. Reacting to these common viral components with neutralizing antibodies would allow the immune system to respond rapidly to infection and potentially serve as a basis for a vaccine. HIV (and its close relative simian immunodeficiency virus [SIV]) avoids antibody neutralization, in part by masking these essential components with flexible structural elements such as sugars and protein domains. The structural variability of the virus forced the authors to combine over a hundred electron micrographs to visualize the structure of the individual virus particles. The authors could then computationally extract the surface components and generate their average structure. This average sheds light on mechanisms of occlusion of common viral components from the immune system. This average structure could serve as a basis for effective vaccine design.

## Introduction

The envelope glycoprotein (Env) complexes of human immunodeficiency virus type-1 (HIV-1) and the related lentivirus simian immunodeficiency virus (SIV) mediate viral entry into their respective host cells. The complex is initially assembled from three copies of the precursor polypeptide gp160, which are cleaved to yield a final mature trimer of heterodimers of the gp120 and gp41 subunits (reviewed in [1]). The surface glycoprotein gp120 confers cellular tropism to the virus by binding CD4 and a co-receptor (primarily CCR5 or CXCR4), and acts as a trigger for the fusogenic activity of the transmembrane glycoprotein gp41. Gp41 associates with gp120 via noncovalent interactions, is anchored into the viral lipid envelope by a transmembrane domain (reviewed in [1]), and contains a C-terminal tail that can be naturally truncated [2].

The current model for immunodeficiency virus entry posits that CD4–gp120 interaction induces and/or stabilizes a conformation in gp120 that allows the exposure and/or creation of previously occluded co-receptor binding surfaces (reviewed in [1]). Co-receptor binding triggers further rearrangement of Env. Based in part upon conserved structural features between influenza hemagglutinin protein (HA) and HIV-1 Env, it has been proposed that co-receptor engagement leading to trimer destabilization acts as a switch for gp41 to assume an extended helical conformation and insert its N-terminus into the target cell membrane (reviewed in [3]). Subsequent refolding of gp41 into a six-helix bundle brings the viral and cellular membranes into apposition, leading to lipid mixing and pore formation.

Env is the target of neutralizing antibodies in infected or immunized hosts because of its location on the virion surface. Several crystal structures of monomeric gp120 cores, lacking the hypervariable loops and N- and C-termini and complexed with specific ligands, have informed our understanding of mechanisms of viral receptor recognition and immune evasion (e.g., [4,5]). More recently, the structures of an unliganded SIV gp120 [6] and a soluble (s)CD4-ligated, HIV-1 gp120 core containing the V3 loop [7] have been determined.

Much of Env function is directed by the protein–protein interactions within the membrane-anchored trimeric spike complex. The structure of this complex has remained unsolved due to its complexity and its instability in solution. Structural studies of intact viruses are limited by the irregular morphology [8] and the low number of surface spikes on lentiviruses [9]. Consequently, many inferences have been made with regard to Env-mediated fusion and neutralizing antibody evasion in the absence of a structure-based molecular model. Recent developments in cryo-electron tomography and image processing make it possible to study the structures of irregular membrane viruses in the native state [10–12]. We have applied these advances to the study of the membrane-anchored spike complex. We have overcome the difficulties related to the low number of spikes by: (1) the use of SIV as opposed to HIV-1, because SIV Env is more stable than its HIV-1 counterpart [13], and is therefore more appropriate for analysis on intact virions, and (2) using an intra-viral tail-truncated form of SIV that contains higher levels of Env than do those viruses with an intact tail. Under these conditions we have collected cryo-tomographic data, and extracted and averaged the spikes to obtain a structure of Env in its native unbound conformation. Fitting of the atomic structure of unliganded SIV Env into this complex has allowed us to propose two models for the functional Env spike. SIV provides a good working model for HIV-1, since the two viruses have a high degree of sequence similarity and exploit CD4 and CCR5 as cellular receptors. These two viruses are also believed to share antibody evasion strategies.

## Results/Discussion

### Tomographic Reconstruction

**Figure 1:**
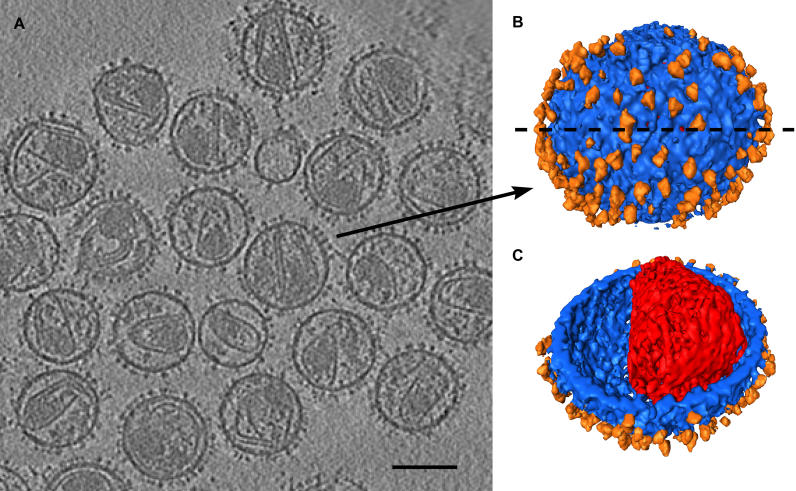
Three-Dimensional Reconstruction of SIV Virions from Cryo-Electron Tomography (A) A slice through the *xy* plane of a reconstructed tomogram from the −6-μm defocus dataset. The spike complexes are clearly visible on the viral surface. Some of the cores appear disrupted, reflecting AT-2 treatment. Scale bar represents 100 nm. (B) Surface rendering of one of the virions. The membrane is represented in blue, the core in red and the spikes in orange. (C) The same virus as in (B) viewed after removing half of the viral envelope along the dotted line to reveal the core. The density between the core and the membrane has not been rendered.

We collected three cryo-tomographic series containing 77 virions. Each had hundreds of spikes visible on the surface (Figure 1), in stark contrast to tomographic reconstructions of NL43 HIV-1 virions [14]. The viral cores were visible, but were not analyzed since we used AT-2 (aldrithiol-2) inactivation which may not preserve native core morphology (J. A. G. Briggs, J. B. Forsdyke, H.-G. Kräusslich, and S. D. Fuller, unpublished data). The SIVmneE11S strain used here has a naturally truncated gp41 intra-viral domain of 17 amino acids [2]. This infectious natural virus variant [15] contains a greater number of Env complexes than SIV containing the full-tail gp41, facilitating tomographic analysis.

The distribution pattern of the spikes appears to be random, with some areas of local ordering (see Figure S1). This ordering may result from a high density of spikes leading to close packing limited by the diameter of the Env complex.

Viral spikes were extracted from the tomographic reconstructions, aligned to one another, and then averaged using an adaptation of the image processing protocol used by Förster et al. [11]. The alignment protocol compensates for the inherent incompleteness of electron tomographic data (the “missing wedge”), which otherwise introduces alignment bias into the reconstruction [11] (see Materials and Methods).

**Figure 2:**
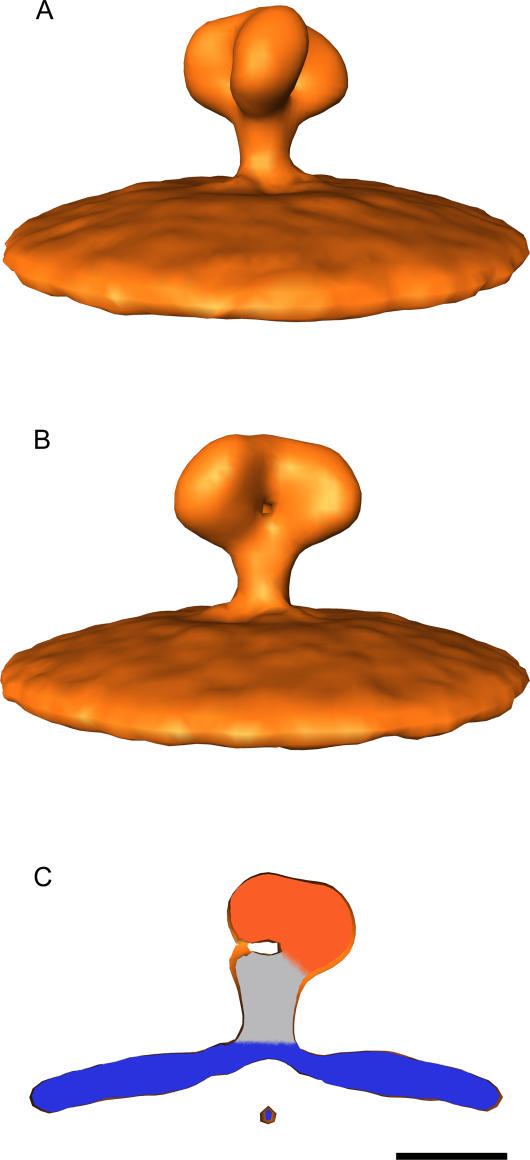
Reconstruction of the Env Spike, Filtered to 28 Å, and Rendered (A) A rendered view of the complex at a 2 sigma contour level. The chirality of the distal portion is evident. (B) As in (A), rotated 60° around the 3-fold symmetry axis. (C) A slab through the density in the orientation represented in panel (B), revealing the cavity at the centre of the structure. The membrane is coloured in cyan. The volume corresponding to gp41 has been coloured in gray, and the remaining gp120 volume in orange. Scale bar = 100 Å.

**Figure 3:**
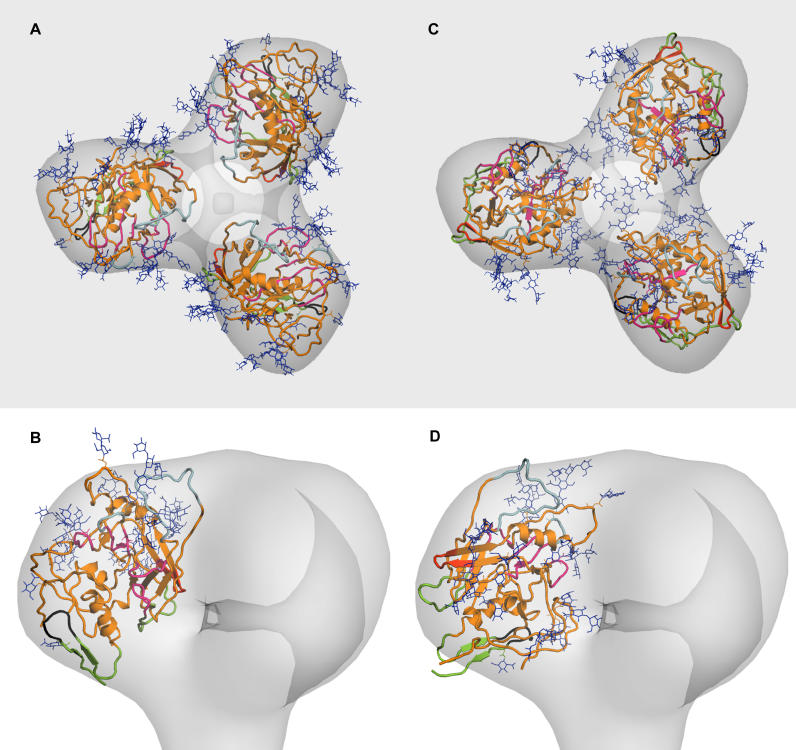
Fitting of the Molecular Model of Unliganded SIV gp120 into the Reconstruction (A) and (B) Views of the first fitting (shown from above in panel [A] and from the side in panel [B]). The variable loops V1V2 (black), V4, and V5 (light blue) are positioned on the surface of the trimer, whereas V3 (red) is closer to the trimer interface. The receptor binding pocket (magenta) is exposed, although partially protected by sugars (blue) and variable loops. The bridging sheet (green) is hidden at the trimer–gp41 interface. Sugar residues are exposed on the surface. (C) and (D) Views of the second fitting (shown from above in panel [C] and from the side in panel [D]). The colour code is the same as in panels (A) and (B). Variable loops V1V2, V3, V4, and V5 are exposed on the surface of the trimer. The receptor binding pocket is also exposed and partially protected by sugars. The bridging sheet is exposed, and the V3 loop could protect it from antibody binding. Sugar residues are exposed.

A total of 2,986 spikes contributed to the final map (Figures 2 and 3, and Video S1). The resolution, based on a conservative 0.5 Fourier shell correlation threshold, is 28 Å. The spike exhibits clear 3-fold symmetry and projects approximately 120 Å from the viral membrane. It consists of a stem approximately 35 Å wide and 50 Å high, capped by three globular densities that appear to fold over the stem in a right-hand propeller orientation. The distance between the tips of the three globular domains is 110 Å. This morphology is strikingly different from that of murine leukaemia virus (MuLV), the only other viral spike reconstructed using this approach [11]. This difference in morphology is not surprising since the surface proteins of the two complexes are unrelated in sequence or structure. MuLV SU is a roughly L-shaped 70 KDa glycoprotein in which seven N-linked glycosylation sites have been identified ([16,17], whereas SIV gp120 core adopts an ovoid structure with 23 potential glycosylation sites [6]). We repeated the alignment procedures using the MuLV spike, and the enantiomer of our final structure as starting models to rule out the possibility of reference bias. In both cases we recovered the same final structure, indicating that our choice of starting model did not bias the alignment. The dimensions of our structure are consistent with molecular modelling with the CD4-bound HIV-1 gp120 crystal structure [18] and with models based on the SIV gp120 core crystal structure [6]. Negative staining of intact virions [9] gave smaller dimensions, probably reflecting artefacts related to the staining and drying of the sample. The volume of the structure, excluding the membrane, is approximately 470 nm^3^ using a 2 sigma contour level.

The gp41 stem appears as a compact structure with no obvious separation between the three monomers. The membrane proximal region of gp41 is characterised by a highly conserved hydrophobic region, which is thought to mediate trimer self-assembly [19]. Our data strongly support this, since we observe a compact stalk, rather than a separation into three legs as for MuLV [11]. Furthermore, the density corresponding to the gp41 stem region is shorter and slightly wider than the post-activation coiled-coil conformation [20], in agreement with the hypothesis that a dramatic conformational change is required for gp41 to extend towards, and insert into, the target cell membrane.

**Figure 4:**
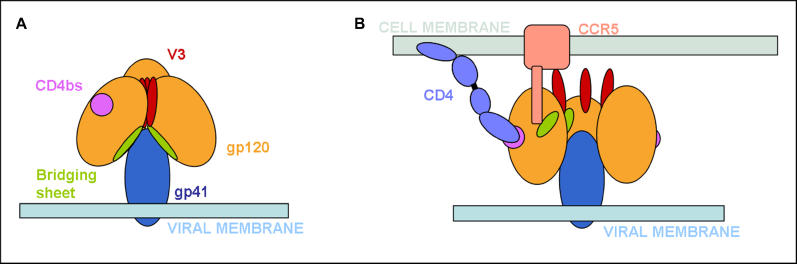
Model for HIV/SIV Receptor Engagement, Based on the First Proposed Fitting (A) The Env complex before CD4 binding. The bridging sheet is not accessible to co-receptor and antibodies, and the V3 loop is at the trimer interface. (B) CD4 binding causes a conformational change and an overall rotation of the gp120 molecule. The V3 loop binds selectively to co-receptor, and the bridging sheet becomes exposed and available for co-receptor binding.

The shape of the complex suggests a new model for the Env trimer organisation. The volumes are consistent with the assignment of the globular domains to gp120 and the stem to gp41. The gp120 protomers appear to fold over gp41 rather than depart radially from it, contacting each other at the top of the spike. The interaction between gp120 monomers at this contact is likely to be weak, since it is not stable in the absence of gp41 [21]. This is consistent with the need for the trimer to disassemble to allow gp41 extrusion (Figure 4). A cavity in the centre of the density, visible at a 2 sigma contour level, suggests that the gp120 trimer interface is separated from the top of the gp41 trimer (Figures 2 and 3).

### Fitting of the Unbound SIV gp120 Core into the Density

Subtracting the total volume of a 28-Å low pass–filtered 44-KDa gp41 ectodomain trimer [20] from the stem region allowed us to approximately assign the remaining density to gp120. We fitted the atomic model of the unbound SIV gp120 core domain into this density. Both the atomic structure and the available density are ellipsoidal, permitting fitting in four orientations. These fits can be assessed in the light of biophysical and biochemical considerations [18]. The CD4 binding surface should be exposed on the surface of the trimer. Carbohydrates should be exposed to solvent because glycosylation sites are almost exclusively present on the exposed surfaces of proteins, and are required on Env for evasion of neutralizing antibodies. Some non-glycosylated, highly conserved sequences are likely to be buried at the trimer interface or hidden behind variable loops. Additionally, the C1 and C5 regions responsible for the interaction with gp41 [22] should be proximal to the gp120–gp41 interface. Based upon these considerations, two fits are consistent with the observed density (Figure 3).

The precision of the fit is limited by the resolution of the tomographic reconstruction, and by the lack of availability of relevant crystal structures. The crystallographic structure of unbound SIV gp120 core lacks the hypervariable loops V1V2 and V3, and the N- and C-terminal domains are also truncated. The missing volume constitutes a significant portion of the protein (a total of 183 residues are absent, about a third of the native protein). The mobility of the variable regions is not known: although they are very flexible in the monomer, they might be partially stabilized in the trimer. Furthermore, the conformation of the protein in the crystal cannot be assumed to reproduce exactly the conformation in vivo. This is particularly true for a protein that is subject to major structural changes and that lacks a significant portion of its structural and functional elements. A rough estimate of the volume of the map suggests that the variable loops are in their majority not visible in the reconstruction. The extent to which sugars are represented in the electron density map is unclear. For these reasons it is not possible to distinguish between the two fits only based upon the shape of the reconstructed density. It may be possible to distinguish the models based on future studies in which gp120 protomers can be more accurately oriented by binding “landmarks,” such as sCD4 or antibodies with known epitopes, to the Env spike of intact virions.

### Interpretation of the Fitted Structures

The two best fits have a number of features in common. In both fitted structures, all glycans are positioned so that they could project outwards from the surface of the trimer, and the variable regions V4 and V5 are positioned on the top surface of the complex, an orientation that would facilitate their recognition by antibodies [23]. The CD4 binding surface is exposed on the outer edge of each gp120 protomer, and is orientated such that access to membrane-associated CD4 on the target cell is possible.

The stem of the V1V2 loop points outwards in both fittings, implying good exposure to solvent. There is evidence that the V1V2 loop impedes antibody access to co-receptor binding sites in monomers of gp120 [24]. A previous structural model suggested that within the trimer, this may result from an interaction between the V1V2 loop and the co-receptor binding site in adjacent monomers [6]. This cannot be excluded in either of our proposed models, although the finding that V1V2 could serve to partially mask the CD4 binding pocket to protect it from antibody recognition [24] suggests that the loop acts on the same monomer to protect conserved regions.

The C1 and C5 regions proposed to bind gp41 are located in the truncated N and C termini of the gp120 protein. The distance of the truncated ends from the gp120–gp41 interface in both our models is consistent with the folding of the missing residues towards gp41. It is also likely that the tip of gp41 diverges outwards from the stem to bind gp120.

The two models we propose differ dramatically in the orientation of the V3 loops and in the position of the co-receptor binding sites. In the first case (Figure 3A and 3B), the V3 base points towards the trimer interface. The density in the reconstruction not occupied by the crystal structure could be attributed to the V3 loop, which would run roughly parallel to the 3-fold symmetry axis, being partially exposed in the cavities between the lobes. In this trimer model, therefore, the V3 loop would be substantially masked by packing into the trimer axis, potentially reinforced by inter-V3 bonding. In the second model (Figure 3C and 3D), the V3 loop is oriented outwards, exposed to solvent. There is no extra density corresponding to the position of V3, meaning that V3 would be highly flexible in the CD4-unbound trimer. The extent of V3 exposure before CD4 binding is dependent on the viral origin and its adaptation to growth in tissue culture. Several studies demonstrate the inability of most V3-specific monoclonal antibodies (mAb) to bind and neutralize primary isolates of HIV-1 [25], and there is little evidence for V3 loop-specific neutralizing antibody activity against SIV, whereas V3 is a target for neutralising antibodies in tissue culture–adapted HIV-1 strains [26]. CD4–gp120 engagement is thought to elicit a conformational change in Env that increases V3 exposure. This is supported experimentally by the increased accessibility of the V3 loop in the Env trimer to antibody binding and enzymic proteolysis subsequent to CD4 engagement [27,28]. It is also consistent with the highly exposed nature of the V3 loop in the structure of the V3-containing gp120–CD4–Fab complex [7]. The increased exposure of V3 in the CD4-bound conformation could be explained by a rearrangement of the trimer in the first model, or by a conformational change involving the V3 loop in the second.

The conserved co-receptor binding site, comprising the bridging sheet [6] and associated regions, is thought to be largely inaccessible in the trimer. Experimental data support this concept, since most CD4-induced (CD4i) surface-specific mAbs cannot access their epitopes on the CD4-unligated Env trimer, as evidenced by weak or absent neutralization by these mAbs [29]. The co-receptor binding region is mostly buried at the trimer interface in our first model, and only a relatively dramatic allosteric change in each gp120 protomer, or a major shift in gp120 orientation, or both, would permit its accessibility to cell surface receptors. Its position in the second model proposed is on the outside of the lobe; in this case the V3 loop could act as a protection from antibody recognition and binding.

A trimer model has been proposed previously in which the inner domain of gp120 points towards the gp120-gp41 interface and the outer domain extends outwards [6]. Such a model is inconsistent with the orientation of the major axis of the ellipsoidal gp120 density within the density corresponding to gp120 in our structure. In this previous model, the V3 loop is exposed to solvent and the co-receptor binding site is buried at the trimer interface. Our two fittings, however, make clear that either the V3 loop is also pointing towards the trimer interface, or the co-receptor binding site is on the outside of the trimer, protected from solvent by V3.

It is likely that the Env complex exhibits some conformational flexibility. Regions such as variable loops and the co-receptor binding site might be transiently exposed and hidden. The high variability in the extent of neutralization of different viral isolates by antibodies against the V3 loop and the ability of certain viruses to bind to co-receptor without the need of prior receptor binding, might reflect the absence of a unique conformation for the assembled trimer. In principle, the map we obtained may average features of more than one conformation of the Env complex. Future work will develop three-dimensional classification tools appropriate to the analysis of structural variability in the glycoprotein spikes.

The two models proposed shed light on several important antigenic and mechanistic features of the Env trimer. First, they provide models consistent with the concept that glycans and immunodominant variable loops are positioned on exposed gp120 surfaces to damp the neutralizing antibody response. Second, they confirm that only a limited gp41 surface is exposed for antibody binding, as has been proposed previously [30]. Third, they provide two possible descriptions of the position of the V3 loop and the co-receptor binding surface in the unbound spike. Finally, by limiting the number of possible gp120 fittings to two, they exclude other possible models for the trimer.

During the review of the work presented here, another study was published reporting the three-dimensional structure of the SIV Env complex in its CD4 unbound conformation [31] obtained using a similar electron tomography based approach. Although the height and breadth of the structures are similar, the shapes of the two structures differ, markedly in the “stem region” of the spike. In contrast with our structure, each monomer in the three-dimensional map obtained by Zhu et al. [31] is multilobed and the transmembrane gp41 glycoprotein divided into three separate densities. The reasons for the differences between the two structures are unclear. We consider it unlikely that the differences between the two structures reflect a difference between the two virus strains. A possibility that we cannot exclude is that the two different reconstructions represent two different conformations of the glycoprotein present on the viral surface selected during the alignment and averaging.

The two studies differ in a number of details of the image processing methodology. Most notably, we have used three distinct starting models and have treated the resolution anisotropy (caused by the “missing wedge” in tomographic data due to the restricted tilt range) during the alignment process [11]. Given the novelty of the image processing procedures used in these studies, a direct and detailed comparison of the two approaches is necessary to rule out the possibility that these methodological differences are responsible for the contrasting structures.

### Conclusions

The three-dimensional reconstruction of the in situ, functional trimeric Env spike is an important step forward for two reasons.

First, visualising the three-dimensional shape of the spike gives insight into potential molecular mechanisms of viral fusion and immune evasion. It suggests the presence of a gp120 trimer interface separated from the gp120–gp41 interface. This implies that disassembly of the receptor binding protein may happen in two steps: disassembly of the gp120 trimer interface followed by separation of gp120 from gp41. The first step could be triggered by CD4 binding and be necessary for co-receptor binding. The first of the two fits that we present could be an elegant explanation of this mechanism, as represented in Figure 4. In the scheme proposed, the CD4-induced rotation of gp120 monomers would simultaneously expose both the V3 loop and the bridging sheet, allowing co-receptor engagement.

Second, the three-dimensional density provides a basis for the fitting of atomic resolution structures, and the assessment of models for trimer structure. Here, these fittings lead to two possible models for the organisation of the CD4-unbound Env trimer, which contribute to advancing the knowledge of the trimeric Env assembly and hence could contribute to the improvement of structure-based drug and vaccine design.

Future studies using defined gp120 ligands will help confirm the correct fitting.

## Materials and Methods

SIVmneE11S particles released into the supernatant by infected HuT 78 cells (SIVmne/Hut 78 CL E11S) were inactivated by treatment with aldrithiol-2, a mild oxidizing agent that does not alter Env function [32], then purified by sucrose gradient centrifugation as described in [33].

Samples were mixed with BSA-adsorbed 10-nm colloidal gold and vitrified for cryo-electron microscopy [14]. Data were collected on a Philips CM300FEG transmission electron microscope (FEI, Eindhoven, The Netherlands), equipped with a GATAN GIF 2002 postcolumn energy filter (Gatan, Pleasanton, California, United States), and images were collected with a 2K × 2K Multiscan CCD camera (Gatan). The microscope was operated at 300 kV and a final magnification of 55,000×, giving a pixel size of 0.55 nm at the specimen level. Tilt series were collected in semi-automatic mode, covering a minimum angular range of 123°, with an angular increment of 3°. Defocus was measured along the tilt axis after each tilt and automatically maintained. Tilt series were collected at defocus values of −6 ± 0.5 μm and −4 ± 0.5 μm, with a total electron dose of between 50 and 70 electrons/Å^2^.

Tilted images were aligned using the gold beads as fiducial markers to within a maximum bead-positioning error of two pixels (11 Å). Three-dimensional reconstructions were obtained using weighted back projections. Alignment, reconstruction, extraction of sub-tomograms, and further image processing were performed using the TOM (http://www.biochem.mpg.de/tom) [34] software packages implemented in MATLAB (Mathworks, Natick, Massachusetts, United States). Visualization was performed with Amira (http://www.amiravis.com) and PyMol (http://pymol.sourceforge.net).

Sub-tomograms (360 pixels) containing individual virions were extracted for use in further processing, as described in [11]. From the −6-μm defocus sub-tomograms, 300 viral spikes were identified by eye, extracted in 63-pixel cubic boxes, aligned, and then averaged [11]. The box size was chosen to contain part of the viral membrane and intraviral material. The averaged structure was used as a template for identification of further spikes by a global cross-correlation search of spherically masked tomograms of individual virions. A missing-wedge function was applied to the Fourier transform of the template so that no preferential orientation of the spikes was selected due to the limits on the extent of the tilted data. The spikes corresponding to the highest 200 cross-correlation peaks for each virus were extracted and manually inspected to remove false positives such as gold beads and particles overlapping with others on neighbouring viruses. A total of 2,115 spikes (from 26 virions) were picked for the −6-μm dataset, and 4,698 (from 51 virions) for the −4-μm. Initial Euler angles [11] were assigned for the −6-μm dataset by assuming the spike was oriented perpendicular to the viral surface and aligning the *z* axis with the vector between the centre of the virus particle and the centre of the extracted spike. Phi, the rotation around this axis, was initially randomized. Fifteen alignment iterations were carried out according to [11] using an ellipsoidal mask. An initial alignment was performed only allowing rotation around the *z* axis. The view along z showed clear 3-fold symmetry after five iterations (Figure S2). Ten further alignment iterations were carried out allowing rotation around all axes, applying 3-fold symmetrisation to the reference. A missing wedge was applied to the Fourier transform of the appropriately rotated references in each alignment before cross-correlation with each particle [11]. Omitting this step can lead to alignment of the particles relative to the missing wedge instead of the particle signal, introducing artefacts into the final structure. An averaged reconstruction was obtained incorporating 1,820 spikes with a cross-correlation threshold set to 80% of the mean cross-correlation value. This reconstruction was then used as a starting model for alignment of spikes from the −4-μm dataset following a similar scheme as used for the −6-μm dataset. The number of spikes incorporated into the reconstruction after each iteration was increased by gradually lowering the cross-correlation coefficient threshold. After over 60% of the dataset was included, the sampling of the angular increment was increased from 5° to 2°, and the overall angular range searched was decreased. Alignments of phi only were performed every three to five complete alignments steps to avoid preferential accumulation of noise. Iterations were continued until the Fourier shell correlation (FSC) curve and the mean cross-correlation coefficient did not improve further. The final reconstruction had a resolution of 28 Å as judged using the 0.5 FSC criterion, and was low-pass filtered to this resolution.

The molecular model of SIV unliganded gp120 was initially manually fitted into the density corresponding to gp120 and then refined using the program URO [35].

## Supporting Information

Figure S1 The Distribution of Spikes Identified and Input into Alignment Protocols The histogram represents the shortest distance between neighbouring spikes picked for the reconstruction (averaging 88 per virion). The average spacing between neighbouring spikes was 137 ± 36.5Å.
(1.8 MB TIF)

Figure S2 Three-Fold Symmetry of the Env Complex Slices on the *xy* plane through the membrane distal region of the Env complex (corresponding to the gp120 trimer). Panel (A) shows the density after one iteration of alignment of the in plane rotation angle phi. Panels (B–F) show the density after two to six iterations of alignment, without imposing any symmetry. The 3-fold character of the Env complex is clear from the output of these early alignment steps.
(2.2 MB TIF)

Video S1 Structure of the SIV Env Complex on the Viral Membrane (1.0 MB WMV)

### Accession Numbers

The European Molecular Biology Laboratory (EMBL)-European Bioinformatics Institute Macromolecular Database (http://www.ebi.ac.uk/msd-srv/emsearch/index.html) accession number of the cryo-electron microscopy map of the SIV spike glycoprotein structure in situ is EMD-1216. The UniProtKB/Swiss-Prot (http://www.ebi.ac.uk/swissprot) accession number for the MULV SU glycoprotein precursor is P03385 and for the SIV envelope glycoprotein precursor gp160 is P05885. The Protein Data Bank (http://www.rcsb.org/pdb) accession number for the unliganded SIV gp120 core is PDB 2BF1.

## Abbreviations

Env: envelope glycoprotein
MuLV: murine leukaemia virus
SIV: simian immunodeficiency virus
