# Supplementary figures and images for: Cryo-Electron Tomographic Structure of an Immunodeficiency Virus Envelope Complex In Situ
Source: PLoS Pathog. 2006 Aug 25;2(8):e83. doi: 10.1371/journal.ppat.0020083 (PMC1557830; doi:10.1371/journal.ppat.0020083)

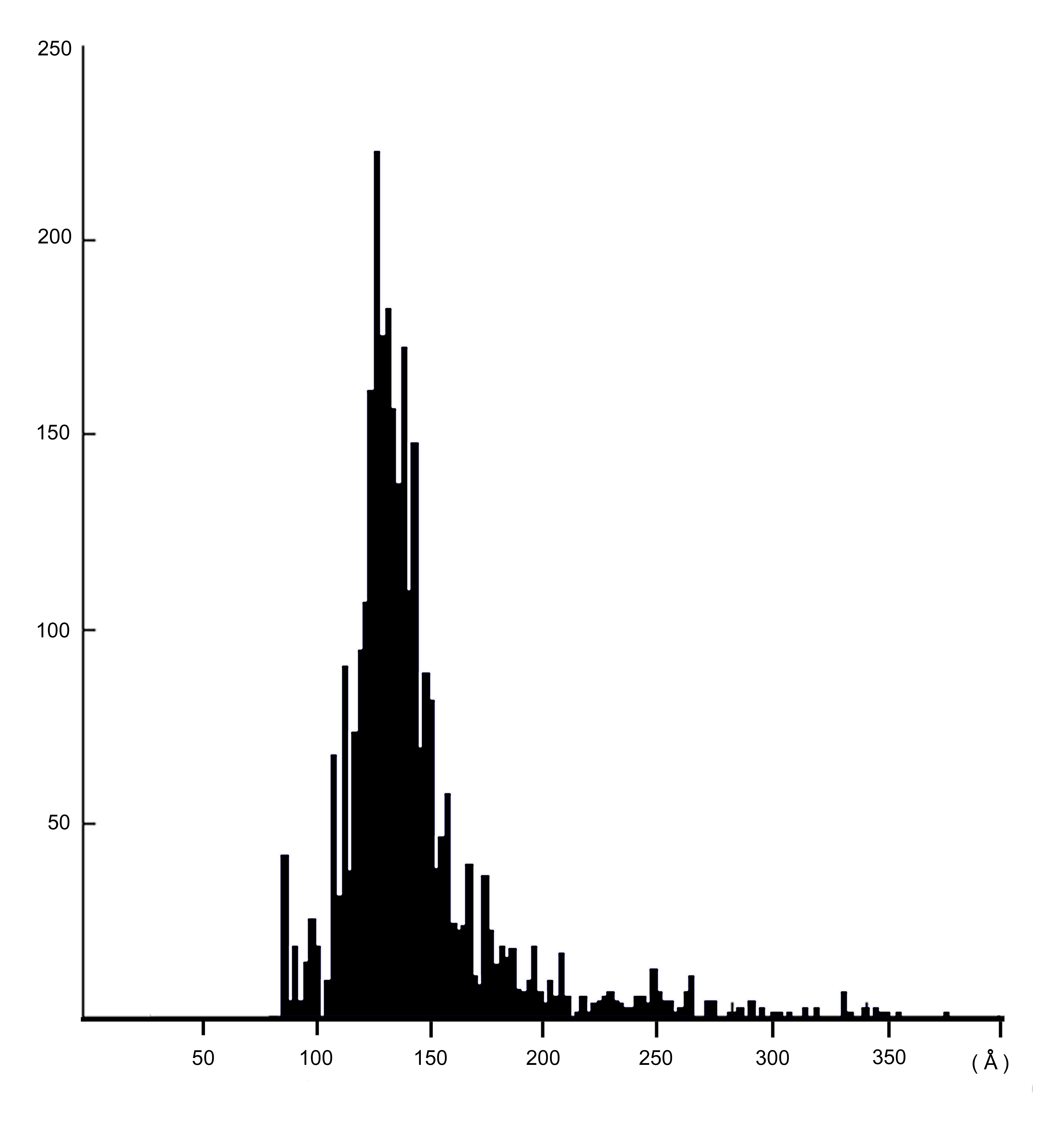

Supplement: Figure S1 — The histogram represents the shortest distance between neighbouring spikes picked for the reconstruction (averaging 88 per virion). The average spacing between neighbouring spikes was 137 ± 36.5Å. (1.8 MB TIF) [file ppat.0020083.sg001.tif]

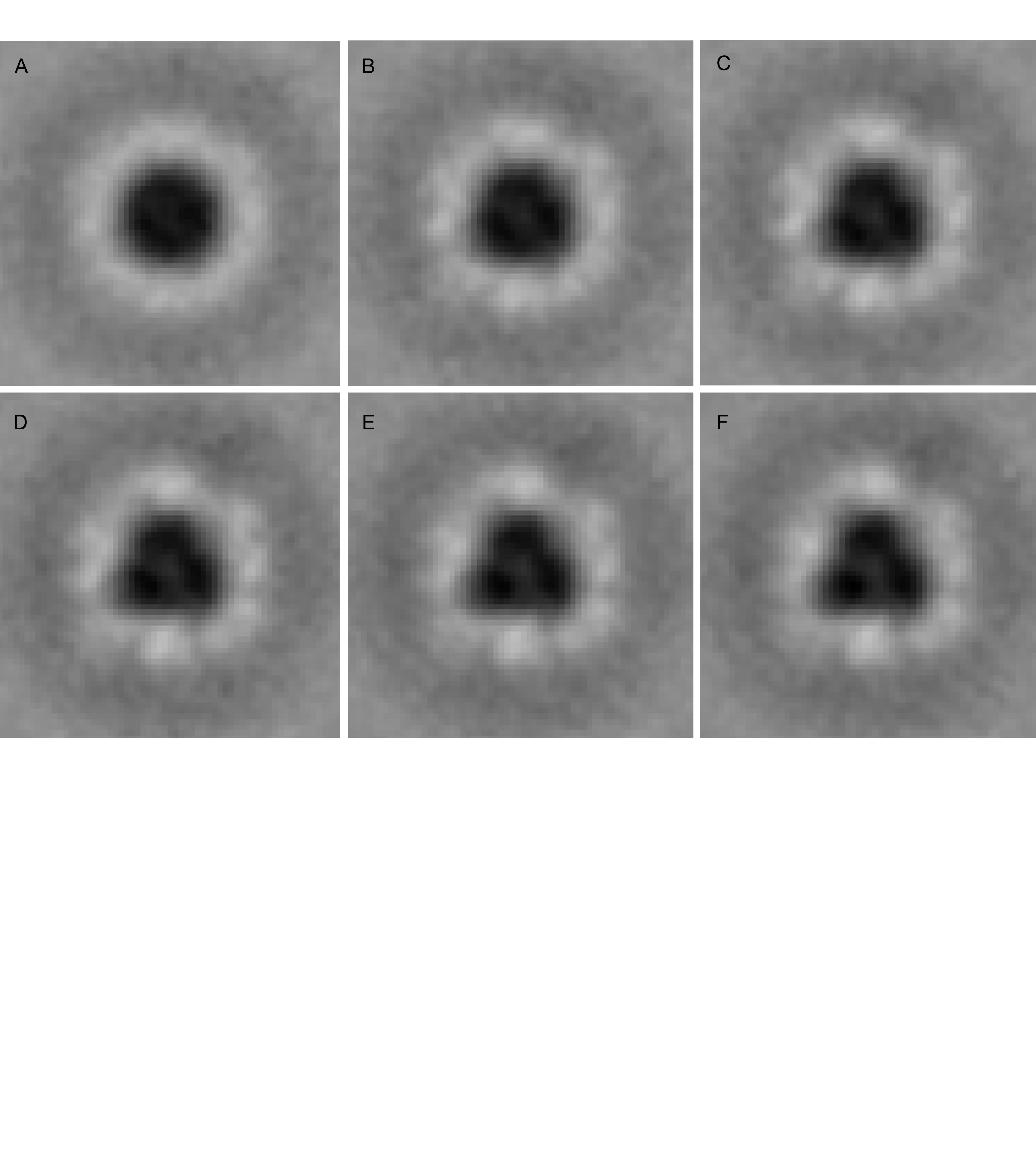

Supplement: Figure S2 — Slices on the xy plane through the membrane distal region of the Env complex (corresponding to the gp120 trimer). Panel (A) shows the density after one iteration of alignment of the in plane rotation angle phi. Panels (B–F) show the density after two to six iterations of alignment, without imposing any symmetry. The 3-fold character of the Env complex is clear from the output of these early alignment steps. (2.2 MB TIF) [file ppat.0020083.sg002.tif]
